# Trypanosoma

**Published:** 1902-07-05

**Authors:** 


### TRYPANOSOMA.

The announcement is made that the Liverpool School of Tropical Medicine is about to send out another expedition to the Gambia to investigate the conditions under which trypanosoma occur in human blood, and to ascertain if possible how these parasites are conveyed from man to man. The matter is one of considerable interest. It had long been supposed that these parasites were confined to the lower animals. Certain species produce the fatal "fly-disease," which is so destructive to domestic animals in South Africa, and the "surra" of horses in India, but in some animals, as for example in the rat, the parasite may occur without producing symptoms. The discovery of typical trypanosoma in the blood of man raises many questions in regard to some of the as yet unclassified fevers of the tropics. Malaria covers by no means the whole field of tropical fevers, and it is quite possible that some of the unhealthiness of the West Coast of Africa may be due to the infection of man by other parasites of which at present we know little, trypanosoma among the rest. Indeed the knowledge that these parasites abound in rats in non-tropical countries, even in England, coupled with the fact that as yet we do not know how they obtain entry to man's blood or under what circumstances, if any, they give rise to symptoms, shows that the proposed investigation is one of very wide interest.

